# A Framework for Prediction of Oncogenomic Progression Aiding Personalized Treatment of Gastric Cancer

**DOI:** 10.3390/diagnostics13132291

**Published:** 2023-07-06

**Authors:** Fahad M. Alotaibi, Yaser Daanial Khan

**Affiliations:** 1 Department of Information System, Faculty of Computing and Information Technology, King Abdulaziz University, Jeddah 21589, Saudi Arabia; 2 Department of Computer Science, University of Management and Technology, Lahore 54770, Pakistan

**Keywords:** long and short-term memory (LSTM), bi-LSTM, gated recurrent units (GRU), next generation sequencing (NGS), gastric carcinoma, deep learning, bioinformatics

## Abstract

Mutations in genes can alter their DNA patterns, and by recognizing these mutations, many carcinomas can be diagnosed in the progression stages. The human body contains many hidden and enigmatic features that humankind has not yet fully understood. A total of 7539 neoplasm cases were reported from 1 January 2021 to 31 December 2021. Of these, 3156 were seen in males (41.9%) and 4383 (58.1%) in female patients. Several machine learning and deep learning frameworks are already implemented to detect mutations, but these techniques lack generalized datasets and need to be optimized for better results. Deep learning-based neural networks provide the computational power to calculate the complex structures of gastric carcinoma-driven gene mutations. This study proposes deep learning approaches such as long and short-term memory, gated recurrent units and bi-LSTM to help in identifying the progression of gastric carcinoma in an optimized manner. This study includes 61 carcinogenic driver genes whose mutations can cause gastric cancer. The mutation information was downloaded from intOGen.org and normal gene sequences were downloaded from asia.ensembl.org, as explained in the data collection section. The proposed deep learning models are validated using the self-consistency test (SCT), 10-fold cross-validation test (FCVT), and independent set test (IST); the IST prediction metrics of accuracy, sensitivity, specificity, MCC and AUC of LSTM, Bi-LSTM, and GRU are 97.18%, 98.35%, 96.01%, 0.94, 0.98; 99.46%, 98.93%, 100%, 0.989, 1.00; 99.46%, 98.93%, 100%, 0.989 and 1.00, respectively.

## 1. Introduction

Gastric cancer is a malignant cancerous mutation disease. It is the 4th most common cancer among men. Mutation is one of the leading causes of this cancer. Mutation is a genetic disorder that occurs due to changes in the gene sequence. These changes may include deletion, insertion, updation or replication of the gene bases in the gene sequences. The American Joint Commission on Cancer (TNM) divided cancer into four stages: 0, 1, 2, 3, and unstageable. In Pakistan, 6566 new cases were identified in 2020, and 5692 deaths were reported. According to Shaukat Khanum Memorial Cancer Hospital and Research Center (SKMCHRC) 7539 new cases were reported from 1 January 2021 to 31 December 2021. Of these, 3156 were seen in males (41.9%) and 4383 (58.1%) in female patients [1]. The smallest component of DNA, the gene, is a two-fold helix particle made up of direct arrangements of nucleotide sets [2]. Each nucleotide is made up of sequence of gene bases. Gene mutation is a type of gene alteration in which the structure of a gene cell is altered. These mutations can provide details about the development of cancer [3]. As researchers gain a deeper understanding of these mutations, the reasons for asymmetrical carcinoma cell proliferation are growing in number. Gastric carcinoma can be recognized using a variety of biomarkers. Even in the absence of physical symptoms on the body or other imaging resources used to detect gastric carcinoma, we can still identify gastric carcinoma by identifying patterns in the TCGA of gene mutations [4]. Different forms of carcinomas can be distinguished by focusing on several types of gene mutation. When a mutation occurs in a person’s body, it accelerates the growth of certain tumor cells, which results in an increase in the number of active gastric carcinoma cells in the body. This alters the normal cycle of cell genesis and apoptosis [5]. Such alteration forces the death process to stop while the body is still producing new cells. Therefore, the increase in the number of cells in the body is termed gastric carcinoma.

This research aims to make significant contributions in the field of gastric cancer mutations by addressing the limitations of the most recent innovative work. The following is the arrangement of the information in bullet form:

- Explore the development of a universal and explicit benchmark dataset specifically tailored for gastric cancer mutations to overcome existing limitations.
- Investigate potential handcrafted feature extraction techniques to preserve the dataset’s integrity and enhance the accuracy of mutation detection models in gastric cancer.
- Examine the shortcomings of current model evaluation methods for accurately assessing the performance of mutation detection models in gastric cancer.
- Propose the development of more robust and comprehensive evaluation techniques to address the limitations of current model evaluation methods.
- Explore the incorporation of improved feature extraction techniques and advanced evaluation methods to enhance the accuracy in the field of gastric cancer mutations.

The proposed study uses the gene sequence dataset for the identification of gastric carcinoma. The most recent and most generalized dataset, as described in the data collection section, was assembled for this study while keeping these limitations in mind. Furthermore, a total accuracy of 99.46% is achieved by utilizing various deep learning methods. Numerous assessments and validation methods are investigated, including the SCT, IST, and 10FCVT. Multiple statistical tools for model evaluation, such as sensitivity, specificity, AUC, and MCC, are also implemented.

## 2. Related Works

The ability to quickly identify cancer using machine learning is advancing every day. Many papers and research articles have been published on various platforms utilizing various methodologies. Most of these studies uses MRI images for the detection of the gastric cancer. As shown in Table 1, machine learning techniques have become widely used in recent years to provide timely identification models for efficient decision making [6,7,8,9,10,11,12,13,14,15,16,17,18,19,20].

Several studies have explored different machine learning techniques for the detection and classification of gastric cancer. A notable approach is the adaptive neural-fuzzy inference system (ANFIS) [6], which achieved an accuracy of 86.00% using PET-Scan and CT-Scan data. Densely connected convolutional networks (DenseNet) [7] demonstrated promising results, with an accuracy of 96.79% when analyzing endoscopy images. Logistic regression [8] was applied to electronic health records and achieved an accuracy of 73.20%. Naive Bayes [9] was utilized for gene expression data, achieving an accuracy of 74.90%. Support vector machines (SVM) [10] achieved an accuracy of 70.00% when analyzing miRNA data.

Furthermore, ensemble learning methods have been employed to improve accuracy. Extra tree classifier, random forest classifier, bagging classifier, and HGB classifier achieved accuracies of 97.27%, 95.64%, 95.21%, and 95.29%, respectively [11]. These classifiers were employed using data from the Surveillance, Epidemiology, and End Results (SEER) database. Other classification algorithms were also explored. The LightGBM (LGBM) classifier achieved an accuracy of 92.71%, while the decision tree classifier achieved 85.75% accuracy. The gradient boost classifier attained an accuracy of 79.54%. Despite the notable performance of these existing approaches, limitations persist.

The most recent innovative works in the field of gastric cancer mutations have encountered various limitations that necessitate attention. One notable constraint is the absence of a universal and explicit benchmark dataset exclusively focused on gastric cancer mutations. To address this, further efforts should be made to incorporate more handcrafted feature extraction techniques that can effectively preserve the integrity of the actual dataset, thereby enhancing the accuracy of mutation detection models. Moreover, the existing model evaluation methods are deemed insufficient, leaving significant room for improvement in terms of accurately assessing the performance of these models. Thus, it becomes imperative to develop more robust and comprehensive evaluation techniques to effectively measure and enhance accuracy in future research endeavors.

## 3. Materials and Methods

**Figure 1:**
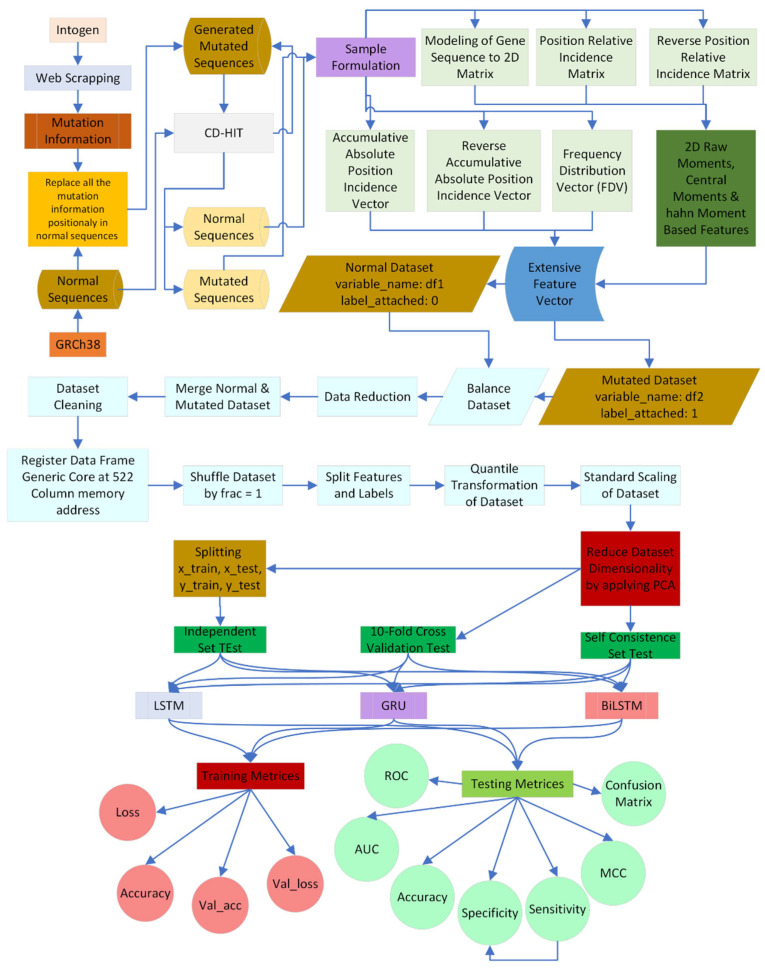
Methodology of the proposed study for identification of mutation to detect stomach carcinoma.

To detect gastric cancer, this study suggests the use of deep learning techniques such LSTM, Bi-LSTM, and GRU. Figure 1 explains this study’s general methodology.

### 3.1. Benchmark Dataset Collection

**Figure 2:**
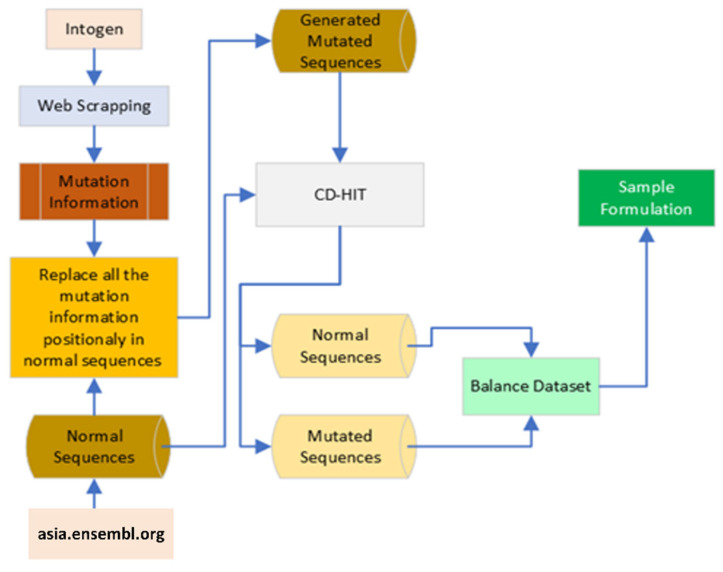
Benchmark dataset collection process.

The benchmark dataset typically includes tentatively settled unambiguous known patterns. These patterns are additionally utilized for testing purposes. Its purpose is to create a high quality benchmark dataset [21,22,23,24,25] which is different, precise, and applicable. There are 1014 samples with 1948 mutations in the 61 driver genes that are connected to gastric cancer. Normal gene sequences were obtained at https://asia.ensembl.org [26] using Python web scraping code, while mutation information were obtained from http://intogen.org [27], also using Python web scraping code. Then, by putting the mutation information into regular gene sequences, another piece of Python code was built to produce mutated sequences. This gives us mutated sequences, but due to the substantial number of mutations and normal dataset, we used the CDHIT tool with a 100% similarity ratio to remove similar sequences from the normal and mutated sequence dataset, leaving us with an unbalanced dataset. Thus, we balanced the dataset to use it for sample formulation [28], and the process is depicted in Figure 2.

This framework requires intense computational power, and one of the most powerful tools available for the lowest cost was Google Colab pro, with 16 GB of GPU and 26 GB of RAM, which took almost 52 h to complete without any interruption. There were a total of 61 gastric carcinoma active mutated driver genes, which are listed in Table 2. All the related driver gene symbols and numbers of mutations in each gene are listed in Table 2.

### 3.2. Feature Extraction

**Figure 3:**
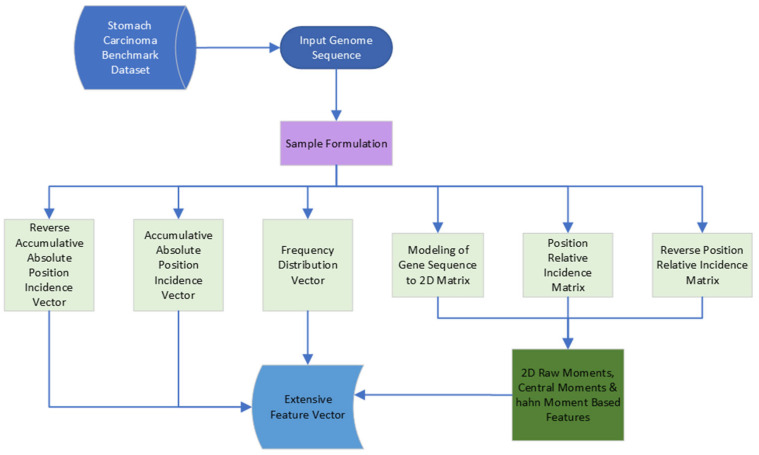
Feature extraction framework.

Redundancy reduction is helpful for deep learning prediction models which specifically include unsupervised learning. This process helps to support complex data structures, i.e., genes mutation data set. After the successful identification of redundant information, data can be compressed. This can reduce the volume of data without losing any valuable information; only scrappy and messy data, which makes the dataset more complex, are eliminated by this procedure [29]. Extensive feature extraction techniques were developed in this study to prepare the dataset for feeding into the proposed deep learning models, as in Figure 3. Multiple feature extraction techniques were applied in this study, such as reverse accumulative absolute position incidence vector (RAAPIV), accumulative absolute position incidence vector (AAPIV), frequency distribution vector (FDV), modeling of gene sequence to 2D matrix, position relative incidence matrix (PRIM), re-verse position relative incidence matrix (RPRIM), 2D raw moments, central moments, and Hahn moments, as discussed in [30,31,32,33,34,35,36,37,38,39,40,41], which required extensive research. Equations (2)–(22) describes all the corresponding elements to extract extensive feature vectors based on int64 datatype, which is ideal for LSTM-based architectures. Intelligible and significant information endures import, since the result obtained is a mixture of various unmistakable fair dataset tests. A sizable dataset with a clear description of the malignant growth driver quality successions is put together [42]. As a baseline of genuine malignant growth driver quality sequences, the dataset is required. This work took the benchmark dataset from a very recent version of the interpretation made accessible on the internet, specifically http://intogen.org/ [43]. A sum of 32 malignant growth driver potential genes mutations, i.e., *TP53*, *CDH1*, *SMAD4*, *KRAS*, *APC*, *KMT2D*, *CDH11*, *ERBB3*, *RHOA*, *LRP1B*, *ARID2*, *BCOR*, *ERBB2*, *KMT2C*, *PTEN*, *FBXW7*, *NIN*, *FAT4*, *PRF1*, *PRKCB*, *RNF43*, *BMPR2*, *SDC45*, *ARHGEF12*, *PIK3R1*, *MYH920*, *NTRK317*, *FAT390*, *BCL914*, *ATM31*, *KIT13*, and *CACNA1D*, are associated with gastric carcinoma-causing mutations [24].

Thusly, information accumulated in this way is utilized to plan a benchmark dataset The benchmark dataset for gastric carcinoma inside the current review is signified as *D,* which is characterized as

(1) $$D=D^{+}\;U\;D^{-}$$

The final benchmark dataset included 1948 carcinoma-mutated human gene sequences ($D^{+}$) and 2000 precisely chosen carcinoma-negative sample genes ($D^{-}$), acquired from a larger collection of normal genes after careful preprocessing and homology reduction. Gene sample presentations often employ two diverse types of model development. Most vector formulations use discrete or sequential modelling to represent genomes. The sequential model uses Equation (2) to represent the genome sequence as its nucleotide sequence:

(2) $$S=w_{1},w_{2},w_{3},\ldots w_{n}$$

where

$$w\in \left\{A\left(adenine\right),C\left(cytosine\right),G\left(guanine\right),T(thymine)\right\}$$

where *w* denotes the nucleotide at any location, and stands for an element contained within the set, with the meaning “member of,” [44], the first nucleotide in genome S is represented by $w_{1}$, and $w_{L}$ is the last nucleotide. ‘*n*’ represents the total length of the sequence in a genome. The detailing of organic sequencing is one of the most basic issues in computational science. The nucleotide makeup of a genomics sample serves as the discrete model representation in the second model. Equation (3) defines the genome S representation using a discrete model as follows:

(3) $$S={\left[{ds}_{1}\;{ds}_{2}\;{ds}_{3}\;\ldots \;{ds}_{20}\right]}^{T}$$

where the useful component feature ${ds}_{a}\left(a=1,2,3,\ldots 20\right)$ is represented by the extraction techniques employing pertinent nucleotides in the genome S. These elements are also used in the statistical moment-based feature extraction techniques.

#### 3.2.1. Statistical Moments Calculation

The arrangement of each succession of genes follows some examples. Because of such requirements, each arrangement is portrayed with various measurable boundaries. In past work, factual moments were utilized for highlight extraction [45,46,47]. To include extraction, crude, focal, and Hahn moments are utilized. The nucleotide component is crucial to the function and makeup of genes. Area and scale variation can be used to extract the component [48]. Crude moments are used to calculate the mean, fluctuation, and imbalance of test appropriation in the dataset in order to address region variation highlights. As mean, difference, and unevenness are assessed using centroid, but focal moments are scaled variably, focal moments are also used for extraction. However, this method is area invariant [29,49]. When measuring measurable limits, Hahn moments are used; however, they come in both area and scale variants [50,51]. In order to evaluate the dataset’s mean, variance, and deviation of the probability transmission, Hahn moments are registered using Hahn polynomials. For the aforementioned method, events are recorded in a n×n two-dimensional grid denoted by $A_{2}D’$ [42]. For portraying the parts and estimations of Equation (4) and the quantitative depiction of gastric carcinoma driver quality, examples of the benchmarks dataset are used in the real methodology.

This study applied factual moments to change the genomics information to a proper size. Every second portrays some novel data that assigns the idea of information. Examiners and mathematicians have dealt with snapshots of various distributions. Hahn, crude, and focal snapshots of the genomics information are outfitted into the list of capabilities and structures as a striking part of an info vector for the indicator. The region and size of fluctuation integrated into the moments can be used as a device to interpret among practically various groupings. The building of a classifier using the distribution of a labeled dataset also benefits from many moments that define the unbalanced and the average of the information. Researchers have discovered that the design, in addition to the general placement of their bases, affects the characteristics of proteomics and genomics arrangements. From this point forward, only mathematical and statistical models are best suited for outfitting the component vector because they are sensitive to the general positioning of component DNA nucleotides inside genomics successions. It is a basic consideration in forming, yielding, and persevering element sets. Since Hahn moments require two-layered information, the genomics groupings are changed into a two-layered documentation A’ of size k * k, which stores a similar amount of data to *S*, though in a two-layered structure to such an extent that

(4) $$m=\sqrt{n}$$

where ‘n’ is the sequence length of a sample genome and ‘m’ represents the 2*D* square matrix dimensions. The $A_{2D}^{\prime }$ matrix in Equation (5) is formed using the ordering obtained from Equation (4), having ‘k×k’ rows and columns, respectively.

A purpose $\omega^{29}$ is a mapping purpose cast-off for matrix transformation of S as $A_{2D}^{\prime }$. It uses the component from this matrix $A_{2D}^{\prime }$. The raw moments are computed using the values of $A_{2D}^{\prime }$. The raw moments of $M_{ij}$, a 2*D* continuous function with the order (i+j), were computed up to order three, such as $M_{01},M_{10},M_{12},M_{21},M_{30}andM_{03}$, and the raw instants are computed as in Equation (6).

(5) $$A_{2D}^{\prime }=\left[\begin{array}{c} \begin{array}{c} a_{1\to 1}\\ a_{2\to 1}\\ {\begin{array}{c} \vdots \\ a \end{array}}_{i\to 1} \end{array}\\ {\begin{array}{c} \vdots \\ a \end{array}}_{k\to 1} \end{array}\begin{array}{c} \begin{array}{c} a_{1\to 2}\ldots \\ a_{2\to 1}\ldots \\ {\begin{array}{c} \vdots \\ a \end{array}}_{i\to 2}\ldots \end{array}\\ {\begin{array}{c} \vdots \\ a \end{array}}_{k\to 2}\ldots \end{array}\begin{array}{c} \begin{array}{c} a_{1\to j}\ldots \\ a_{2\to j}\ldots \\ {\begin{array}{c} \vdots \\ a \end{array}}_{i\to j}\ldots \end{array}\\ {\begin{array}{c} \vdots \\ a \end{array}}_{k\to j}\ldots \end{array}\begin{array}{c} \begin{array}{c} a_{1\to k}\\ a_{2\to k}\\ {\begin{array}{c} \vdots \\ a \end{array}}_{i\to k} \end{array}\\ {\begin{array}{c} \vdots \\ a \end{array}}_{k\to k} \end{array}\right]$$

(6) $$W_{ij}=\sum_{b=1}^{n}\sum_{q=1}^{n}b^{i}q^{j}A_{2D}^{\prime }(b,q)$$

The order of the instants is indicated by the addition of i and j, that is, i+j, which can be less than or equal to three. The above equation’s raw moments are computed up to the third order. The origin of the data is used as the starting point from which these raw moments are computed and as the measurement of the separation between the components [45]. The unique characteristics of the raw moments were computed as $W_{00},W_{01},W_{10},W_{11},W_{02},W_{20},W_{12},W_{21},W_{30}\;and\;W_{03}$. The centroid of any piece of data is also thought to be its center of gravity. an information point from which the information is evenly spread in all directions. The relationships shown here are those of its weighted average [52]. Using the centroid of the data as their reference point, the central moments unique feature is computed from Equation (7) up to the third order.

(7) $$Q_{ij}=\sum_{b=1}^{n}\sum_{q=1}^{n}{\left(b-\bar{x}\right)}^{i}{\left(q-\bar{y}\right)}^{j}A_{2D}^{\prime }(b,q)$$

The unique features from central moments, up to the third order, are labeled as $Q_{00},Q_{10},Q_{01},Q_{11},Q_{02},Q_{20},Q_{12},Q_{21},Q_{30}\;and\;Q_{03}.$ Here, the centroids are calculated as $\bar{x}$ and $\bar{y}$ from Equations (8) and (9):

(8) $$\bar{x}=\frac{M_{10}}{M_{00}}$$

(9) $$\bar{y}=\frac{M_{01}}{M_{00}}$$

Hahn instants can be easily computed for an even-dimensional data body. Reversible possessions of Hahn instants are manifest due to their orthogonality. The square network is utilized as the discrete contribution to figure Hahn moments. Hahn moments assist with depicting the evenness of information and, simultaneously, they are reversible. This essentially implies that these moments can be utilized to reproduce the first information. The reversibility of moments guarantees that the data shortened inside the first arrangement stays in a salvageable shape and is passed forward to the indicator through the relating highlight vector. Hahn moments are processed utilizing Equation (10), for any integer $r\in \left[0,P-1\right](P\;is\;a\;given\;positive\;integer)$. Hahn instants or order n are computed as

(10) $$h_{n}^{u,v}\left(r,P\right)={\left(P+v-1\right)}_{n}{\left(P-1\right)}_{n}\times \sum_{k=0}^{n}{\left(-1\right)}^{k}\frac{{\left(-n\right)}_{k}{\left(-r\right)}_{k}{\left(2P+u+v-n-1\right)}_{k}}{{\left(P+v-1\right)}_{k}{\left(P-1\right)}_{k}}.\frac{1}{k!}$$

where ${\left(a\right)}_{k}=a\left(a+1\right)\ldots \left(a+k-1\right)=\frac{\Gamma (a+k)}{\Gamma (a)}$ is the Pochhammer symbol and u,v(u>−1,v>−1) control the shape of polynomials. A square matrix $A^{\prime }$ is necessary to express the Hahn moments because of its orthogonal features, which necessitate two-dimensional input data. Equation (10) makes use of the Pochhammer documentation, which in turn makes use of the Gamma administrator. Equation (11) explains the Pochhammer symbol:

(11) $${\left(\Pi \right)}_{s}=\Pi \left(\Pi +1\right)\ldots (\Pi +k-1)$$

The Gamma operator is used to simplify as given in Equation (12):

(12) $${\left(\Pi \right)}_{s}=\frac{\Gamma (\Pi +k)}{\Pi (\Pi )}$$

The raw values of Hahn moments given in Equation (13) are often scaled using a weighting function and square norm:

(13) $$h_{n}^{\tilde{u},v}\left(r,P\right)=h_{n}^{u,v}\left(r,P\right)\sqrt{\frac{\Pi \left(r\right)}{k_{n}^{2}}},n=0,1,\ldots ,P-1$$

Meanwhile, in Equation (14):

(14) $$\Pi \left(r\right)=\frac{{\Gamma (x+r+y)\Gamma (y+r+1)(x+y+r+1)}_{P}}{\left(x+y+2r+1\right)n!\left(P-r-1\right)!}$$

Equation (15) computes the Hahn moments up to third order for the 2D discrete data as follows:

(15) $$H_{xy}=\sum_{q=0}^{P-1}\sum_{b=0}^{P-1}{A^{\prime }}_{ij}h_{x}^{\tilde{u,v}}\left(j,P\right)h_{y}^{\tilde{u,v}}\left(i,P\right),x,y=0,1,\ldots P-1$$

For every genome sequence, 10 raw, 10 central, and 10 Hahn moments are computed, up to the third order, and are further unified into the collection comprehensive feature vector. These unique features are represented by $H_{00},H_{01},H_{10},H_{11},H_{12},H_{21},H_{20},H_{02},H_{30}\;and\;H_{03}$.

#### 3.2.2. Determination of Position Relative Incident Matrix (PRIM)

In next-generation sequencing [53], there are many situations in which the gene arrangements are homologous. This normally happens when a similar predecessor is important for the advancement cycle and more than one grouping is developed from it [54]. In such cases, the exhibition of the classifier is infinitely influenced by utilizing these homologous groupings [55]. Any genome sequence’s nucleotide’s relative location is regarded as a fundamental pattern that makes use of the physical characteristics of the genome sequence. The genomic sequence is represented by the PRIM in $\left(20\times 20\right)$ order. When managing the results, successful and responsible arrangement resemblance looking is carried out in order to produce correct results. The relative position of each nucleotide in the given genome sequence is extracted in the form of a matrix, where $Q_{i\to j}$ contains the accumulated worth of jth buildup as for the underlying Equation (16) of the ith buildup.

(16) $$Q_{PRIM}=\left[\begin{array}{c} \begin{array}{c} Q_{1\to 1}\\ Q_{2\to 1}\\ {\begin{array}{c} \vdots \\ Q \end{array}}_{i\to 1} \end{array}\\ {\begin{array}{c} \vdots \\ Q \end{array}}_{k\to 1} \end{array}\begin{array}{c} \begin{array}{c} Q_{1\to 2}\ldots \\ Q_{2\to 1}\ldots \\ {\begin{array}{c} \vdots \\ Q \end{array}}_{i\to 2}\ldots \end{array}\\ {\begin{array}{c} \vdots \\ Q \end{array}}_{k\to 2}\ldots \end{array}\begin{array}{c} \begin{array}{c} Q_{1\to j}\ldots \\ Q_{2\to j}\ldots \\ {\begin{array}{c} \vdots \\ Q \end{array}}_{i\to j}\ldots \end{array}\\ {\begin{array}{c} \vdots \\ Q \end{array}}_{k\to j}\ldots \end{array}\begin{array}{c} \begin{array}{c} Q_{1\to 20}\\ Q_{2\to 20}\\ {\begin{array}{c} \vdots \\ Q \end{array}}_{i\to 20} \end{array}\\ {\begin{array}{c} \vdots \\ Q \end{array}}_{k\to 20} \end{array}\right]$$

These results represent a replacement of the biological evolutionary process carried out by nucleotides of type “j”. A total of 20 native nucleotide occurrences and positional values are shown in alphabetical order. Successful calculations from position relative occurrences in the form of Q_PRIM provide 400 coefficients. The 2D Q_PRIM matrix was used to compute 10 Hahn moments, 10 central moments, and 10 raw moments up to 3rd order. Additional 30 distinct features were added before feature extraction.

#### 3.2.3. Determination Reverse Position Relative Incident Matrix (RPRIM)

In AI, exactness and productivity are massively subject to the carefulness and painstakingness of calculations through which the most appropriate provisions in the information are extracted. During the learning stage in AI calculations, learning and transformation of the most implanted obscure patterns in the information are performed to disguise the applicable and relevant elements [47,52,55,56]. RPRIM and PRIM calculations have a similar methodology, yet just RPRIM works with the reverse gene sequence requesting. Processing RPRIM reveals stowed-away patterns that empower the justification of any ambiguities between homologous groupings. It is described by Equation (16). Information is extracted as 400 coefficients for PRIM, which produces a set of 24 elements. Likewise, the above approach is utilized to develop and switch PRIM for a similar succession in a contrary application. The RPRIM is given as $Q_{RPRIM}$:

(17) $$Q_{RPRIM}=\left[\begin{array}{c} \begin{array}{c} P_{1\to 1}\\ P_{2\to 1}\\ {\begin{array}{c} \vdots \\ P \end{array}}_{i\to 1} \end{array}\\ {\begin{array}{c} \vdots \\ P \end{array}}_{k\to 1} \end{array}\begin{array}{c} \begin{array}{c} P_{1\to 2}\ldots \\ P_{2\to 1}\ldots \\ {\begin{array}{c} \vdots \\ P \end{array}}_{i\to 2}\ldots \end{array}\\ {\begin{array}{c} \vdots \\ P \end{array}}_{k\to 2}\ldots \end{array}\begin{array}{c} \begin{array}{c} P_{1\to j}\ldots \\ P_{2\to j}\ldots \\ {\begin{array}{c} \vdots \\ P \end{array}}_{i\to j}\ldots \end{array}\\ {\begin{array}{c} \vdots \\ P \end{array}}_{k\to j}\ldots \end{array}\begin{array}{c} \begin{array}{c} P_{1\to 20}\\ P_{2\to 20}\\ {\begin{array}{c} \vdots \\ P \end{array}}_{i\to 20} \end{array}\\ {\begin{array}{c} \vdots \\ P \end{array}}_{k\to 20} \end{array}\right]$$

where $P_{i\to j}$ collected worth of jth buildup concerning the underlaying appearance of the ith buildup utilizing the opposite essential succession. The 2D $Q_{RPRIM}$ matrix was used to compute 10 Hahn moments, 10 central moments, and 10 raw moments up to the third order. The collection of feature extraction was further coordinated to include 30 additional unique features.

#### 3.2.4. Frequency Distribution Vector (FDV)

A frequency distribution vector was created using the distribution of occurrence in each nucleotide of a genomics sequence. Equation (18) defines the frequency distribution vector as follows:

(18) $$\theta =\left\{\varphi_{i},\ldots \varphi_{20}\right\}$$

Here, the occurrence frequency of $ith\left(1\leq i\leq 20\right)$ relevant nucleotide is represented as $\varphi_{i}$. However, these techniques are used to reduce information regarding the position importance of nucleotides in a sequence. Additionally, the collection of feature extraction is further coordinated to incorporate 20 features from a frequency distributed vector.

#### 3.2.5. Accumulative Absolute Position Incidence Vector (AAPIV)

Nucleotide distributional information is stored in the frequency distribution vector, but no information on the relative positions of the nucleotides is pertinent. Using AAPIV, 20 relevant nucleotides in a genomic sequence with 20 associated important features might accommodate relative positioning information [48,57]. The collection of feature extraction also coordinates these 20 essential AAPIV traits as shown in Equation (19).

(19) $$AAPIV=\left\{\beta_{i},\ldots \beta_{20}\right\}$$

Here, $\beta_{i}$ is from genome sequence $R_{x}$ having ‘n’ total nucleotides, which can be calculated using Equation (20):

(20) $$\beta_{i}=\sum_{x=1}^{n}R_{x}$$

#### 3.2.6. Reverse Accumulative Absolute Position Incidence Vector (RAAPIV)

The calculations for RAAPIV and AAPIV follow identical steps; however, only RAAPIV uses the reverse genome sequence ordering. By concealing the deep and hidden patterns of each sample feature, the computing of RAAPIV makes use of reverse relative positioning information [52,58]. The following Equation (21) gives rise to RAAPIV, which produces 20 significant characteristics. These 20 distinct key features from RAAPIV are coordinated with the feature extraction data set.

(21) $$RAAPIV=\left\{\beta_{i},\ldots \beta_{20}\right\}$$

Here, $\beta_{i}$ is from genome sequence $R_{x}$ having ‘n’ total nucleotides, which can be calculated using Equation (22):

(22) $$\beta_{i}=\sum_{x=1}^{n}{Reverse(R}_{x})$$

After features were extracted using the feature extraction approach, 150-D features were created to be used for further processing in the classification algorithm.

### 3.3. Classification Algorithms

LSTM, GRU, and bi-directional LSTM are deep learning algorithms used in this study. These are also explained in the following subsections.

#### 3.3.1. Long Short-Term Memory (LSTM)

Vanishing gradient problems are solved by applying some specific gates in an RNN, which are built as specified in LSTM and commemorated as ℶ, explained in Equation (23):

(23) $$ℶ=\sigma \left({Wx}^{<t>}+{Ua}^{<t-1>}+b\right)$$

where W,U,b are coefficients specific to the gate and σ is the sigmoid function. The update gate ${ℶ}_{u}$ defines how much the past should matter, and is used in LSTM. The reset gate ${ℶ}_{r}$ describes how much previous information should be dropped and is used in LSTM, the forget gate ${ℶ}_{f}$ defines if a cell should be erased or not and is used in LSTM, and the output gate ${ℶ}_{o}$ defines how much to reveal of a cell used in LSTM, applying all modification as Equations (24)–(26).

(24) $$c^{<t-1>}=tanh(W_{c}\left[{ℶ}_{r}\;*\;a^{<t-1>},\;{ℶ}_{f}\;*\;a^{<t-1>},x^{<t>}\right]+b_{c})$$

(25) $$c^{<t>}={ℶ}_{u}\;*\;{\tilde{c}}^{<t>}+{ℶ}_{u}\;*\;c^{<t-1>}+{ℶ}_{f}\;*\;c^{<t-1>}$$

(26) $$a^{<t>}={{ℶ}_{o}\;*\;c}^{<t>}$$

The sign ∗ denotes element wise multiplication between two vectors.

**Figure 4:**
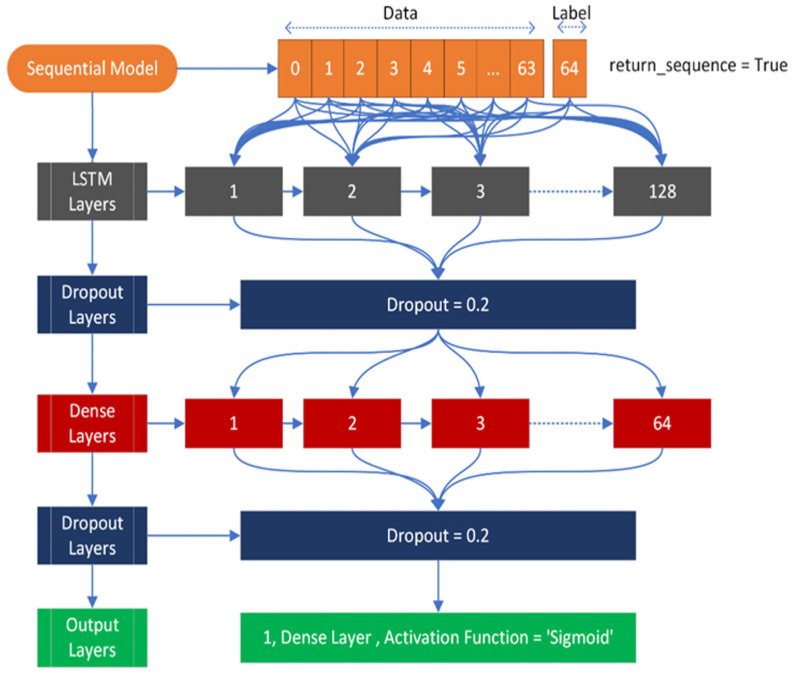
Applied LSTM architecture used to classify mutated and normal gene sequences related to gastric carcinoma.

The input shape is (64, 1), where 64 represents the number of feature fields, and 1 represents the target field, which can be either positive or negative. During compilation, the loss is calculated using binary cross entropy with the Adam optimizer. The model architecture consists of an input layer followed by an LSTM layer with 128 neurons. After the LSTM layer, there are two dropout layers, a dense layer with 64 nodes, and finally an output layer. Both dropout layers will deactivate 20% of the nodes to avoid overfitting. The output layer has one node with a sigmoid activation function. Figure 4 provides a visual representation of this architecture.

#### 3.3.2. Gated Recurrent Units (GRU)

The GRU is a more advanced and simple version of the LSTM that was first developed by [59] for application to machine translation. The GRU is based on the LSTM and controls information flow within the unit via update gate ${ℶ}_{u}$ and Reset gate ${ℶ}_{r}$ without the use of separate memory. As a result, the GRU can capture the mapping connection between time series data [60], and it also has attractive characteristics such as reduced complexity and an efficient computing procedure, which demonstrates the link between the update and reset gates. The update gate ${ℶ}_{u}$ defines how much past should matter which is used in the GRU. The reset gate ${ℶ}_{r}$ describes how much previous information should be dropped and used in the GRU, while the output gate ${ℶ}_{o}$ defines how much to reveal of a cell used in the GRU, applying all modification as Equations (27)–(29):

(27) $$c^{<t-1>}=tanh(Wc\left[{ℶ}_{r}\;*\;a^{<t-1>},x^{<t>}\right]+b_{c})$$

(28) $$c^{<t>}={ℶ}_{u}{\tilde{c}}^{<t>}+\left({1-ℶ}_{u}\right)\;*\;c^{<t-1>}$$

(29) $$a^{<t>}=c^{<t>}$$

**Figure 5:**
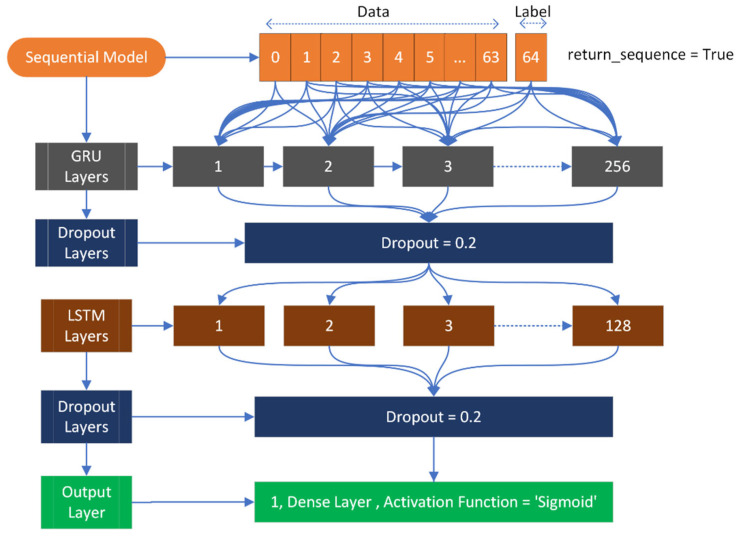
Applied GRU architecture used to classify mutated and normal gene sequences related to gastric carcinoma.

The input shape is (64, 1), where 64 represents the number of feature fields, and 1 represents the target field, which can be either positive or negative. During compilation, the loss is calculated using binary cross entropy with the Adam optimizer. The model architecture includes a GRU layer with 256 nodes, followed by a dropout layer. After the dropout layer, an LSTM layer with 128 nodes is used. The LSTM layer is again followed by a dropout layer, where 20% of the neurons are deactivated to prevent overfitting. Finally, an output layer with a sigmoid activation function is used. Figure 5 provides a visual representation of this architecture.

#### 3.3.3. Bidirectional LSTM (Bi-LSTM)

The learning rate scheduling parameter of the LSTM model is tuned using the Adam optimizer. For each variable in the training process, the learning rate is determined adaptively [61]. Adaptive learning rates for various parameters are computed using the first and second moments of gradients. This variant of stochastic gradient descent is known as Adam by the authors.

Nonlinear sigmoidal gates regulate one or more memory cells in a memory block. These gates control whether the model preserves the values at the gates (i.e., the gates evaluate to 1) or discards them (i.e., the gates evaluate to 0). The network computes a mapping sequence to the output $y=(y_{1}......y_{T})$ given the input sequence $x=(x_{1}......x_{T})$.

Equation (30) can be used to illustrate the fact that information only spreads in the forward direction in LSTM networks, indicating that the state at time t solely depends on the information available before t.

(30) $$\vec{a^{<t>}}=LSTM\left(x^{<t>},\vec{a^{<t+1>}}\right)$$

and when an LSTM backpropagates from the forward direction, then it means direction will be propagated from the last element of the tensor, which can be expresses as Equation (31):

(31) $$\overset{↼}{a^{<t>}}=LSTM\left(x^{<t>},\overset{↼}{a^{<t+1>}}\right)$$

Finally, the output of the bi-LSTM can be summed as Equation (32) by combining the forward and backward states.

(32) $$a^{<t>}=\left[\vec{a^{<t>}},\overset{↼}{a^{<t>}}\right]$$

**Figure 6:**
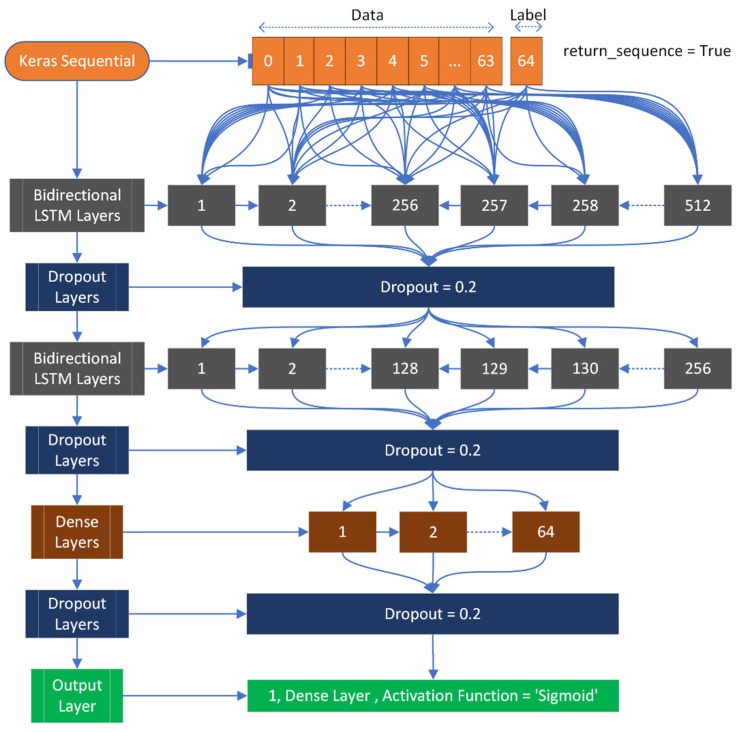
Applied Bi-LSTM architecture used to classify mutated and normal gene sequences related to gastric carcinoma.

The input shape is (64, 1), where 64 represents the number of feature fields, and 1 represents the target field, which can be either positive or negative. During compilation, the loss is calculated using binary cross entropy with the Adam optimizer. The model architecture consists of two Bi-LSTM layers, with the first layer having 512 nodes and the second layer having 256 nodes. To avoid overfitting, three dropout layers are used. Additionally, a dense layer with 64 nodes is included. The output layer is a dense layer with a sigmoid activation function to prevent overfitting. Figure 6 provides a visual representation of this architecture.

## 4. Results

To measure the performance of the suggested prediction model, it is necessary to compare all the results obtained in this study. A comparison of all the results acquired by this study is shown in Table 3.

### 4.1. Self-Consistency Test (SCT)

**Figure 7:**
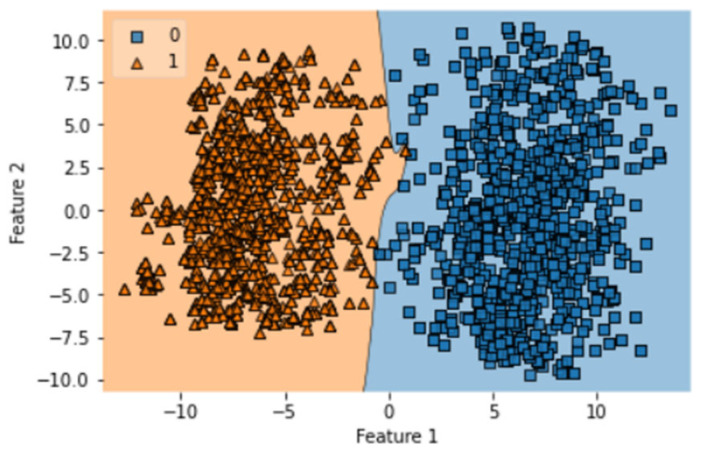
Decision boundary of SCT of LSTM.

After complete evaluation, we identified that GRU is best optimized on one notch benchmark dataset. The obtained accuracy, sensitivity, specificity, MCC, AUC of GRU in ISTs are 99.46%, 98.93%, 100%, 0.989, and 1.00, respectively. The obtained result validates the accuracy of the prediction model. This test requires that the indicator is tried with similar examples which were utilized to prepare it. Hereafter, every one of the classifiers prepared on the benchmark dataset is tried. The quantity of tests accurately anticipated by every one of the classifiers is organized to determine the exactness measurements as displayed in Table 3. Thus, the ROC bend shows an examination of precision displayed by every indicator. It is shown that the exhibition of the bi-LSTM indicator is genuinely flourishing when contrasted with GRU and LSTM. Every one of the outcomes yielded by the depicted test is displayed in Table 3. It demonstrates that the predicted rule that was applied during the evaluation was similar to the first computational method that was suggested for the review. The execution of the many different proposed structures that are concerned with this investigation and the evaluation is also demonstrated. Both the training and testing procedures were coordinated with the same dataset in the SCT, because we already know the true positive rate of our benchmark dataset. This test validates the accuracy of training of formulated prediction model. This model does not provide any robust evaluation in the manner of K-fold cross-validation but still has importance in the overall validation process. The results of SCT are given in Table 3. It can be observed that LSTM, Bi-LSTM, and GRU have accuracy values of 97.18%, 98.88%, and 98.88%, respectively. The AUC obtained by LSTM, Bi-LSTM, and GRU is 0.98, 1.00, and 1.00. It validates the correctness of the GRU and Bi-LSTM classifiers. SCT of LSTM model was completed in 63.39 s with a training accuracy of 97.77%. The decision boundary of SCT of LSTM is shown in Figure 7.

**Figure 8:**
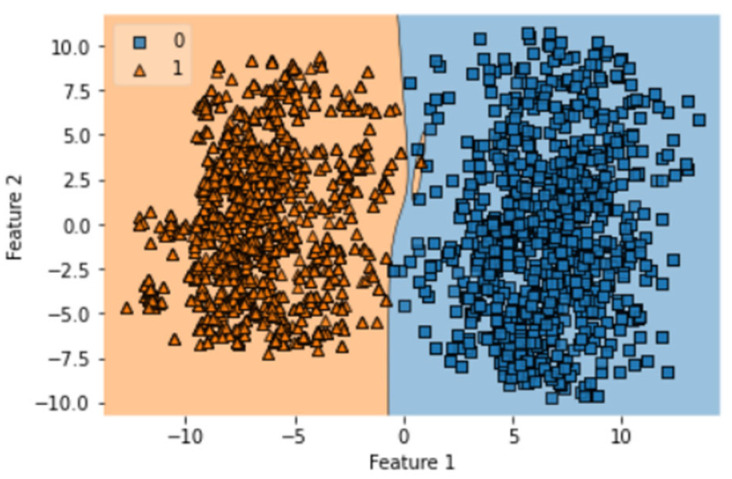
Decision boundary of SCT of GRU.

There are a total of 100 epochs used to fit the LSTM model in which loss decreased simultaneously from 0.68 to 0.097 in SCT. It shows the compatibility of the dataset with the classifier, and an AUC value of 0.98 shows the optimization of this algorithm on one. There are a total of 100 epochs used to fit the GRU model notch benchmark dataset of gastric carcinoma. The decision boundary of SCT of GRU is shown in Figure 8.

**Figure 9:**
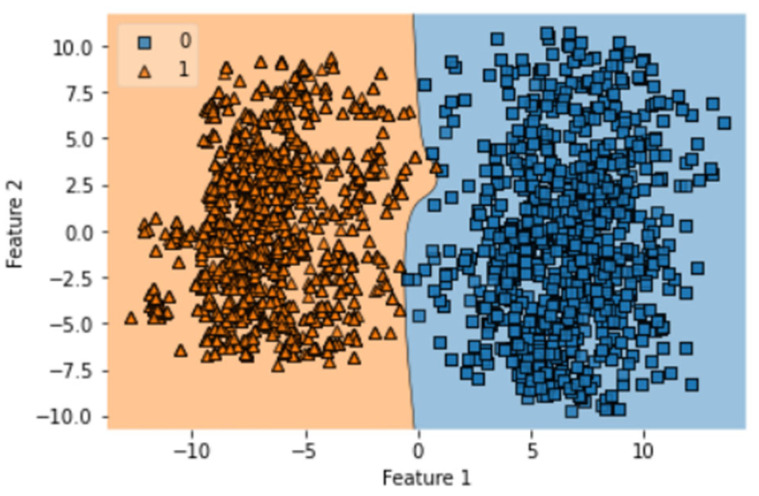
Decision boundary of SCT of Bi-LSTM.

**Figure 10:**
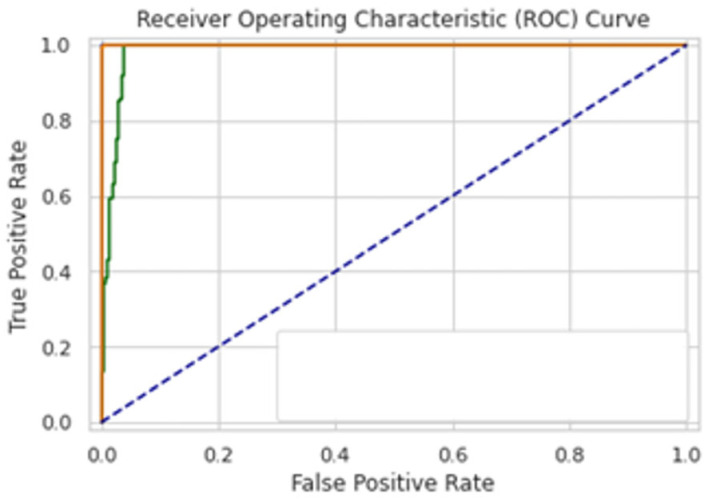
Combined ROC Curve for self-consistency set test of LSTM, GRU, Bi-LSTM.

A total of 100 epochs were used to fit the model for SCT of GRU feature extracted dataset in which loss decreased simultaneously. Moreover, accuracy matrices also increased exponentially, i.e., 53.50 to 100. This behavior shows the exactness of the classifiers with the one-notch benchmark dataset. A total of 100 epochs were used to fit the model with SCT of Bi-LSTM on feature extracted dataset in which loss decreased simultaneously. Moreover, accuracy matrices also increased exponentially, i.e., 92.50 to 100. The decision boundary of SCT of Bi-LSTM is shown in Figure 9. The combined ROC curve of LSTM, GRU and Bi-LSTM is shown in Figure 10. In Figure 10, the green ROC curve illustrates the performance of GRU, while the orange ROC curve represents the performance of Bi-LSTM. The blue dashed line serves as the baseline in the ROC curve, indicating the performance of a random classifier or a model with no discrimination capability.

### 4.2. Independent Set Test (IST)

**Figure 11:**
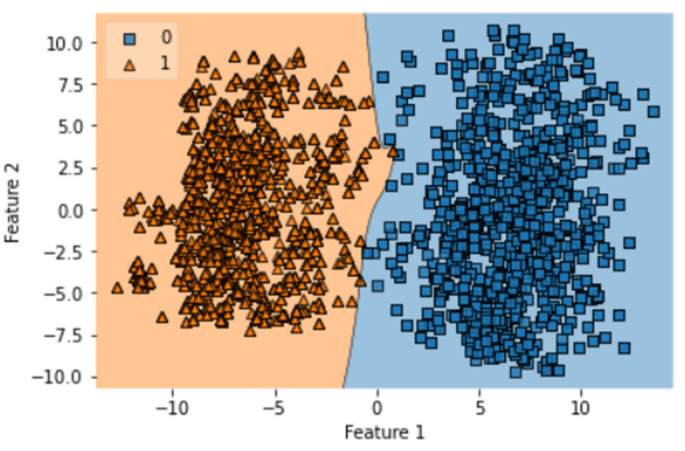
Decision boundary of IST of LSTM.

A total of 100 epochs were used to fit the model with IST of LSTM on feature extracted dataset in which loss decreased. Moreover, accuracy matrices also increased to 97.77%. The decision boundary of IST of LSTM is shown in Figure 11.

**Figure 12:**
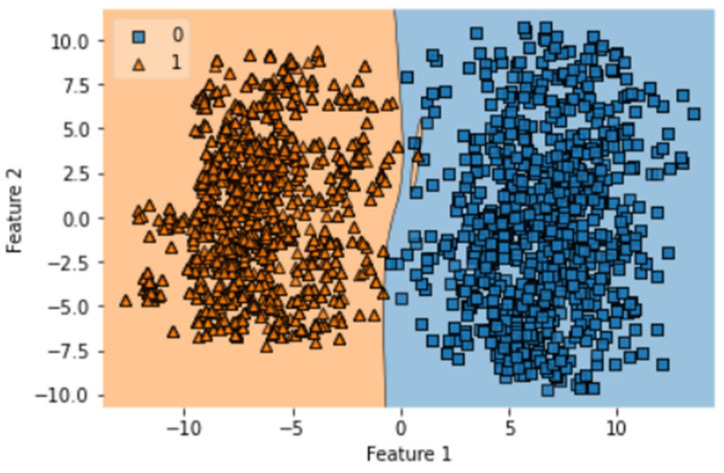
Decision boundary of IST of GRU.

A total of 100 epochs were used to fit the model for IST of GRU on feature extracted dataset in which loss decreased. Moreover, accuracy matrices also increased from 55.67 to 100. The decision boundary of IST of GRU is shown in Figure 12.

**Figure 13:**
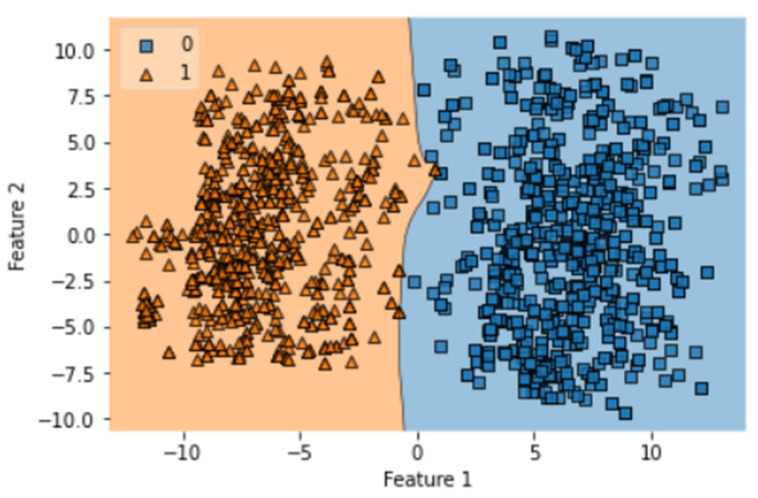
Decision boundary of IST of Bi-LSTM.

**Figure 14:**
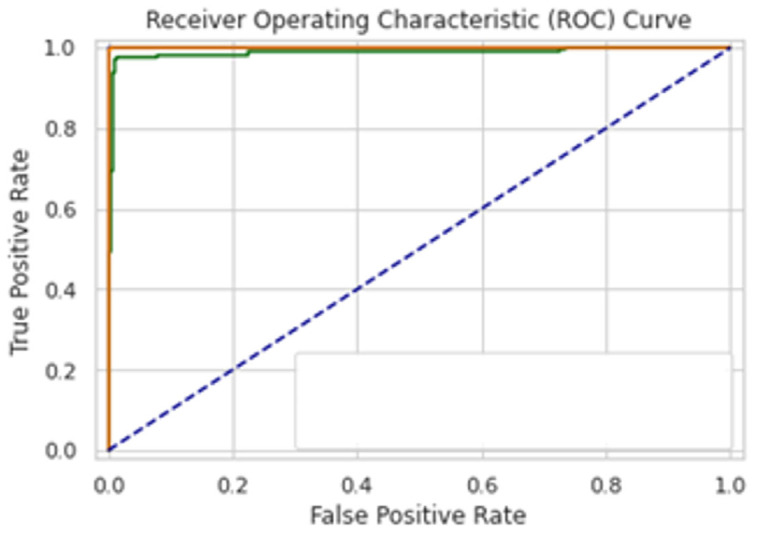
Combine ROC Curve FOR Self-Consistence Set LSTM, GRU, Bi-LSTM.

A total of 100 epochs were used to fit the model for IST of Bi-LSTM on feature extracted dataset in which loss decreased. Moreover, accuracy matrices also increased from 99 to 99.82. The decision boundary of IST of Bi-LSTM is shown in Figure 13. The Combined ROC curve is shown in Figure 14. In Figure 14, the green ROC curve illustrates the performance of GRU, while the orange ROC curve represents the performance of Bi-LSTM. The blue dashed line serves as the baseline in the ROC curve, indicating the performance of a random classifier or a model with no discrimination capability.

### 4.3. 10-Fold Cross-Validation Test (FCVT)

**Figure 15:**
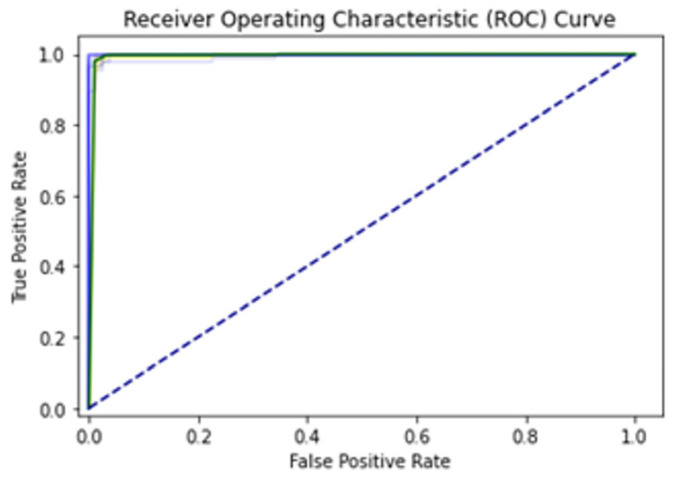
ROC for 10-fold cross validation test of LSTM.

**Figure 16:**
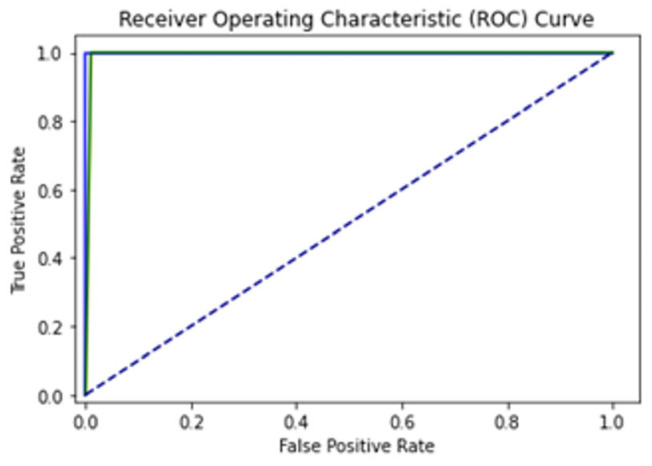
ROC for 10-fold cross validation test GRU.

**Figure 17:**
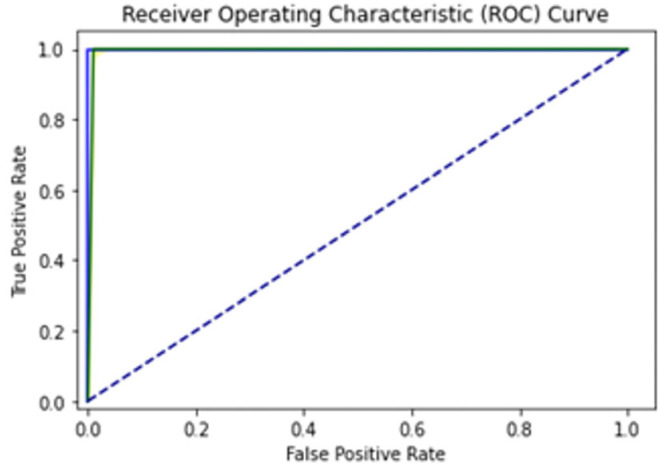
ROC for 10-fold cross validation test Bi-LSTM.

The 10-FCVT sampling test uses a limited number of data samples to validate the formulated prediction model. It has a single parameter, k, which defines how the data sample should be divided. K can be any numeric value; we use k = 10, which folds the overall learning into 10 folds. This it is the best method of validation that predicts true positives. In every fold, a random subset of data is selected for validation from the entire dataset, and accuracy, sensitivity, specificity, and MCC are measured with the mean average value of all fold’s results. Detailed results of the 10-FCVT are given in Table 3. It can be observed that the LSTM, Bi-LSTM, and GRU have accuracy values of 97.30%, 97.89% and 97.83%, respectively. The Mean ROC (MROC) values of the LSTM, Bi-LSTM, and GRU are 0.99, 0.99 and 0.99, and given in Figure 15, Figure 16 and Figure 17, respectively. In Figure 15, Figure 16 and Figure 17, the green ROC curve illustrates the performance of GRU, while the blue ROC curve represents the performance of LSTM. The blue dashed line serves as the baseline in the ROC curve, indicating the performance of a random classifier or a model with no discrimination capability. 

### 4.4. Comparison with Previous Studies

The independent set test results of LSTM, GRU and Bi-directional LSTM are compared with previous studies in Table 4.

In this study, three different deep learning models were developed, namely LSTM, GRU and Bi-LSTM, and achieved peak accuracies of 97.18, 99.46 and 99.46, respectively. It is clear in Table 4 that this study produced better results than the previous results.

### 4.5. Complexity Study

A complexity study was conducted to evaluate the impact of incorporated feature extraction techniques developed in this study. Table 5 presents a comparison of the results obtained using feature extraction techniques developed in this study versus the results obtained without utilizing feature extraction techniques developed in this study.

## 5. Analysis and Discussion

For the identification and detection of gastric cancer, several biological and computational studies have been conducted. Most researchers used sparse datasets from a small number of hospitals or institutions in previous research, applying machine learning algorithms for detection with lower accuracy and fewer assessment matrices. The most recent generalized huge dataset was employed in deep learning, which included LSTM, BI-LSTM, and GRU for the detection of gastric cancer. The collection includes 1948 mutations in 1014 samples from 61 driver genes related to gastric cancer. The most recent and generalized dataset for the normal and mutant gene sequences of gastric cancer is utilized in this study. Other sorts of mutations are also the subject of a study comparable to this one [62,63], and certain testing methods are also discussed in [64,65]. SCT, IST and 10-FCVT are three separate testing procedures that are applied to the dataset accordingly. It can be inferred from the outcomes of the testing procedures indicated above that the suggested models are most suited to attaining high accuracy for cancer prediction. The entire dataset was used for both the training and testing rounds of the SCT. The results are displayed in Table 3. A total of 80% of the dataset was utilized for training and 20% was used for testing in the IST. The outcomes of ensemble learning utilizing an IST are displayed in Table 3. Ten equal folds were produced from the entire dataset for the 10-FCVT. The proposed deep learning models underwent repeated training on 9-folds and testing on 10-fold. For testing and training, the complete set of data is used. For improved learning, scrambled data are presented each time, and then the average is determined. The best accuracies were produced by GRU, such as 98.88%, 99.46%, and 97.89% in SCT, IST and 10-FCVT, respectively. Multiple statistical tools for mode evaluation are used in this study. Sensitivity, specificity, MCC, and AUC obtained through GRU in independent tests were 98.93%, 100%, 0.989 and 1.00.

## 6. Conclusions

This study proposes a framework for identifying the progression of gastric carcinoma by analyzing gene mutations using deep learning-based neural networks. The framework utilizes three RNN variant classifiers: Bi-LSTM, GRU, and LSTM, which were trained on a feature extracted benchmark dataset consisting of 522 fields with labels of either 0 or 1.

The performance and efficiency of the defined models were analyzed using various evaluation metrics, including accuracy, sensitivity, specificity, MCC, and AUC. The results, as presented in Table 3, show that all three models (LSTM, Bi-LSTM, and GRU) achieved high prediction accuracy across different evaluation methodologies (SCT, IST, and 10-FCVT). The metrics demonstrate the models’ ability to accurately identify gastric carcinoma progression, with values ranging from 96.01% to 100% for accuracy, 96.10% to 99.46% for sensitivity, 96.55% to 100% for specificity, 0.94 to 0.989 for MCC, and 0.977 to 1.00 for AUC.

These results highlight the potential of deep learning approaches, specifically the proposed framework using RNN variants, in identifying and predicting the progression of gastric carcinoma. However, it is important to note that further optimization and refinement of these strategies and frameworks are necessary to improve their overall performance and achieve even better results.

This study focused on the important task of mutation detection for early detection of gastric cancer. Our work has aimed to contribute to society by addressing the limitations in the current approaches and proposing novel methodologies to enhance the accuracy and efficiency of mutation detection in gastric cancer. The potential impact of our research is significant. Early detection of gastric cancer can significantly improve patient outcomes and survival rates. By accurately identifying and characterizing genetic mutations associated with gastric cancer, our work can contribute to the development of more precise diagnostic tools and targeted therapies. This can lead to earlier intervention, personalized treatment approaches, and improved prognoses for patients. However, it is important to acknowledge the limitations of our work. We recognize that our proposed methodologies may still have room for improvement and require further validation on larger and diverse datasets. Additionally, the complexity of genetic mutations and the heterogeneity of gastric cancer present ongoing challenges in achieving perfect accuracy in mutation detection.

### Future Work

Future work in this field should focus on dataset expansion to include more diverse samples, improving the feature extraction process through investigation and optimization, exploring alternative deep learning architectures for model refinement, considering additional evaluation metrics for a comprehensive assessment of model performance, conducting external validation on independent datasets, and collaborating with medical professionals for clinical translation. These efforts will contribute to the continuous improvement of computational methods for identifying and predicting the progression of gastric carcinoma, leading to earlier detection, enhanced treatment strategies, and improved patient outcomes.

## Figures and Tables

**Table 1:** Previous applied algorithms to investigate gastric carcinoma.

Paper Citation	Algorithm	Accuracy Achieved	Dataset
[6]	Adaptive Neural-Fuzzy Inference System	86.00%	PET-Scan, CT-Scan
[7]	Densely Connected Convolutional Network	96.79%	Endoscopy Images
[8]	Logistic Regression	73.20%	Electronic Health Record
[9]	Naive Bayes	74.90%	Gene Expression Data
[10]	Support Vector Machine	70.00%	miRNA
[11]	Extra Tree Classifier Random Forest Classifier Bagging Classifier HGB Classifier LGBM Classifier Decision Tree Classifier Gradient Boost Classifier	97.27% 95.64% 95.21% 95.29% 92.71% 85.75% 79.54%	Surveillance, Epidemiology and End Results (SEER)

**Table 2:** All genes related to stomach cancer with the number of mutations in each gene.

Gene Symbol	No of Mutations	Gene Symbol	No of Mutations	Gene Symbol	No of Mutations
*TP53*	293	*FBXW7*	16	*ARHGEF12*	13
*ARID1A*	76	*MAP2K7*	22	*PIK3R1*	5
*PIK3CA*	75	*SOHLH2*	15	*MYH9*	20
*CDH10*	52	*NIN*	18	*NTRK3*	17
*SMAD4*	35	*FAT4*	126	*FAT3*	90
*KRAS*	37	*PRF1*	15	*BCL9*	14
*APC*	44	*PRKCB*	14	*ATM*	31
*KMT2D*	45	*ACVR2A*	24	*KIT*	13
*CDH11*	33	*RNF43*	16	*CACNA1D*	18
*ERBB3*	28	*BMPR2*	11	*KDM6A*	11
*RHOA*	27	*PPP3CA*	9	*CARS*	8
*CTNNB1*	30	*CASP8*	6	*GRIN2A*	32
*LRP1B*	169	*TOP2A*	12	*NSD1*	21
*ARID2*	27	*PRRX1*	9	*FAT1*	31
*CDKN2A*	18	*ARHGEF10L*	10	*CDK12*	15
*BCOR*	28	*TET1*	23	*FHIT*	3
*ERBB2*	26	*RELA*	9	*BCLAF1*	20
*DCSTAMP*	18	*RB1*	12	*RECQL4*	11
*TRIM49C*	17	*NRG1*	23	*CLIP1*	10
*KMT2C*	69	*BMPR1A*	3		
*PTEN*	20	*SDC4*	5		

**Table 3:** Comparison of all the obtained results of this study of LSTM, GRU, and bi-directional LSTM.

Self-Consistency Set Test				Independent Set Test			10-Fold Cross Validation Test		
Metrics	LSTM	GRU	Bi-LSTM	LSTM	GRU	BI-LSTM	LSTM	GRU	Bi-LSTM
Accuracy (%)	97.18	98.88	98.88	97.18	99.46	99.46	97.30	97.89	97.83
Sensitivity (%)	98.35	100	100	98.35	98.93	98.93	96.10	96.67	96.55
Specificity (%)	96.01	97.77	97.77	96.01	100	100	98.56	99.16	99.16
MCC	0.94	0.977	0.977	0.94	0.989	0.989	0.946	0.978	0.978
AUC	0.98	1.00	1.0	0.98	1.00	1.00	0.99	0.99	0.99

**Table 4:** Comparison of the current studies with the previous studies.

Current Study		Previous Studies	
Algorithms	Accuracies Obtained	Algorithms	Accuracies Obtained
LSTM	97.18	Adaptive Neural-Fuzzy Inference System [6]	86.00%
GRU	99.46	Densely Connected Convolutional Network [7]	96.79%
Bi-LSTM	99.46	Logistic Regression [8]	73.20%
		Naive Bayes [9]	74.90%
		Support Vector Machine [10]	70.00%
		Random Forest Classifier [10]	95.64%
		LGBM Classifier [11]	92.71%
		Decision Tree Classifier [11]	85.75%
		Gradient Boost Classifier [11]	79.54%

**Table 5:** A complexity study to assess the contribution of feature extraction techniques developed in this study.

Obtained Results Using Feature Extraction Techniques Developed in This Study		Obtained Results without Using Feature Extraction Techniques Developed in This Study	
Algorithms	Accuracies Obtained	Algorithms	Accuracies Obtained
LSTM	97.18	LSTM	90.42
GRU	99.46	GRU	91.55
Bi-LSTM	99.46	Bi-LSTM	92.67

## Data Availability

Not applicable.
